# No evidence that a transmissible cancer has shifted from emergence to endemism in Tasmanian devils

**DOI:** 10.1098/rsos.231875

**Published:** 2024-04-17

**Authors:** Maximilian R. Stammnitz, Kevin Gori, Elizabeth P. Murchison

**Affiliations:** ^1^ Transmissible Cancer Group, Department of Veterinary Medicine, University of Cambridge, Cambridge, UK

**Keywords:** Tasmanian devils, transmissible cancer evolution, devil facial tumour disease, cancer genomics, computational biology, reproducibility

## Abstract

Tasmanian devils are endangered by a transmissible cancer known as Tasmanian devil facial tumour 1 (DFT1). A 2020 study by Patton *et al*. (*Science* **370**, eabb9772 (doi:10.1126/science.abb9772)) used genome data from DFT1 tumours to produce a dated phylogenetic tree for this transmissible cancer lineage, and thence, using phylodynamics models, to estimate its epidemiological parameters and predict its future trajectory. It concluded that the effective reproduction number for DFT1 had declined to a value of one, and that the disease had shifted from emergence to endemism. We show that the study is based on erroneous mutation calls and flawed methodology, and that its conclusions cannot be substantiated.

## 1. Introduction

Tasmanian devils (*Sarcophilus harrisii*), carnivorous marsupials endemic to the Australian island of Tasmania, are endangered by a transmissible cancer known as Tasmanian devil facial tumour 1 (DFT1, also known as devil facial tumour disease, DFTD) [1–3]. This contagious cancer is spread between devils by the transfer of living cancer cells during biting, and manifests as malignant facial tumours [4,5]. DFT1 first emerged in a single ‘founder devil’ in the 1980s, and its clonal evolution can be represented by a phylogenetic tree [6,7]. The disease has spread through almost the entire Tasmanian devil population, and rapid reductions in devil density driven by DFT1 led to the species being listed as Endangered on the International Union for Conservation of Nature Red List in 2008 [3,8,9]. The rate of population decline has decreased with time, however, and recent modelling studies have predicted the species’ persistence at low density [9–11].

Pathogen genomes can be used to analyse the evolution and epidemiology of infectious diseases [12,13]. A study published in 2020 by Patton *et al*. [14] used DFT1 genomes to characterize the phylogenetic structure of the DFT1 epizootic and to quantify rates of transmission. It concluded that DFT1 had no detectable spatial structure and that its effective reproduction number had declined to a value of one, suggesting a transition to endemism. In separate studies by our group [6,7], we analysed similar but independent datasets and reached different conclusions to Patton *et al*. [14]. This led us to evaluate the data and analysis presented by Patton *et al*. [14]. Here, we outline the results of this reanalysis.

**Figure 1.**
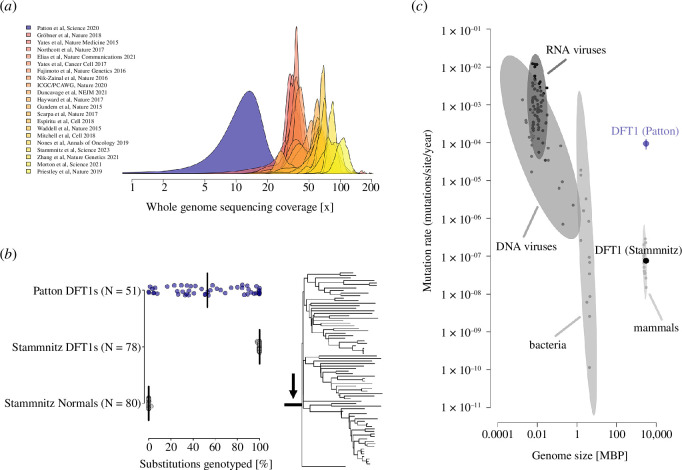
Insufficient genome coverage and flawed mutation calling in Patton *et al*. [14]. (*a*) Whole genome sequencing depth for 51 DFT1 tumours described in the study by Patton *et al*. [14] plotted alongside equivalent data from 20 recent cancer genomics studies targeted towards comprehensive discovery of somatic mutation. Data and references are available in electronic supplementary material, tables S1 and S5. (*b*) 1311 substitutions occurring at the trunk of the DFT1 phylogenetic tree, and thus common to all DFT1s and absent from all studied normal Tasmanian devil genomes, were genotyped in the DFT1 tumours presented by Patton *et al*. [14] and by Stammnitz *et al*. [7]. Each dot represents a tumour or normal genome. Proportion of substitutions detected at a threshold of three reads is displayed, with bar indicating median. DFT1 phylogenetic tree described by Stammnitz *et al*. [7] is shown, arrow indicating the trunk. Data are available in electronic supplementary material, table S2 and data S1. (*c*) Mutation rates observed in various classes of microbes and in mammalian somatic tissues. DFT1 mutation rate estimates from Patton *et al*. [14] and Stammnitz *et al*. [7] are indicated as bold points, with bars representing 95% Bayesian credible intervals. Mammalian and DFT1 mutation rate estimates have not been corrected for the doubling of available sites that occurs with diploidy. MBP, megabases. Data are available in electronic supplementary material, table S6.

Patton *et al*. [14] analysed short-read whole genome sequences from 51 DFT1 tumours. Most of these genomes, however, were sequenced to an unsuitable depth for substitution mutation detection. A minimum of 30× coverage is usually recommended for short-read whole genome sequencing studies targeted towards comprehensive somatic substitution mutation discovery in cancer [15,16]. Upon realigning data from Patton *et al*. [14], we calculated that these tumours were sequenced to a median depth of only 15× (figure 1*a*, electronic supplementary material, table S1). Furthermore, after accounting for tumour purity, the proportion of DNA in each tumour biopsy attributable to cancer cells, the median sequencing depth of actual DFT1 DNA was 9× (eletronic supplementary material, table S1). We quantified mutation detection sensitivity in this tumour cohort by genotyping 1311 substitutions universally present in 78 DFT1 tumours sequenced to a median coverage of 83× and analysed in a separate study by our group [7]. Considering that these variants must also be present in all the DFT1 genomes presented by Patton *et al*. [14] (see below), our analysis suggests a median false negative genotyping rate of 47% (range 0–100%) among the tumour cohort of Patton *et al*. [14] (figure 1*b*, electronic supplementary material, table S2, data S1).

Despite the unsuitability of their data, Patton *et al*. [14] attempted somatic substitution variant calling. This was undertaken using *bcftools mpileup* and *bcftools call* [17], and variants subsequently also identified in any one of 12 normal Tasmanian devil genomes were removed as potential germline contaminants. Patton *et al*. [14] did not provide any further detail on variant quality filtering, nor a list of the final substitutions themselves. Despite the significant expected false negative rate, the mutation frequency reported by Patton *et al*. [14] among 51 tumours within a restricted genomic interval was notably high (5406 variants in 431,608 bp; 1.25 × 10^–2^ mutations per site). This frequency was more than two orders of magnitude higher than that which we identified in the corresponding genomic interval, relative to a new reference genome [7], in 78 DFT1 tumours analysed alongside matched host genomes using standard practice cancer variant calling (22 substitutions in 670‌,660 bp; 3.28 × 10^–5^ mutations per site; electronic supplementary material, table S3). Indeed, the DFT1 mutation rate reported by Patton *et al*. [14] (9.44 × 10^–5^ mutations/site/yr, 95% Bayesian credible interval 6.74 × 10^–5^–1.23 × 10^–4^) is comparable to that reported in some viruses (figure 1*c*), and is implausible for most mammalian cancers. Using our own data, we estimated a DFT1 mutation rate of 7.22 × 10^–8^ mutations/site/yr (95% Bayesian credible interval 7.11 × 10^–8^–7.32 × 10^–8^) [7]. We consider that the most likely explanation for these discrepancies is that the mutations identified by Patton *et al*. [14] are in the majority spurious calls resulting from flawed variant detection and filtering.

**Figure 2.**
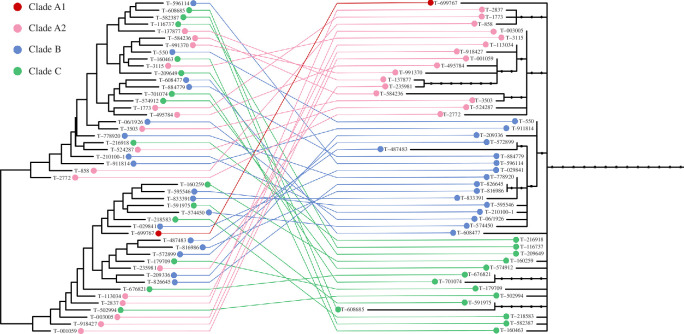
Internal inconsistency in phylogenetic tree topologies among DFT1 tumours presented by Patton *et al*. [14]. Forty-eight DFT1 tumours from Patton *et al*. [14] are displayed on two phylogenetic trees, with lines connecting the equivalent tumour on each tree. Left, maximum likelihood phylogenetic tree, as presented by Patton *et al*. [14], constructed using substitution mutations. Right, maximum likelihood phylogenetic tree inferred using 148 copy number variants (CNVs); small black dots represent 32 phylogenetically informative CNVs (internal branches) and 13 CNVs common to all DFT1s (trunk branch); branch lengths are proportional to number of CNVs. Each tumour’s colour refers to its genotype-assigned DFT1 clade [6,7] (electronic supplementary material, table S2). Three tumours were excluded from both trees as they had insufficient sequencing coverage, after accounting for tumour purity, for CNV inference. CNV data are available in electronic supplementary material, table S4 and data S2.

A phylogenetic tree constructed from erroneous data would be expected to be devoid of biological meaning. Consistent with this, the phylogenetic tree reported by Patton *et al*. [14] suggests that DFT1 shows no spatial structure and that its spread required implausible migration rates. Our studies of DFT1, on the other hand, have revealed that DFT1 split early into at least six monophyletic clades, each showing distinctive spatio-temporal distribution [6,7]. We genotyped the tumours analysed by Patton *et al*. [14] using a panel of several hundred informative somatic substitution mutations, and assigned each to a DFT1 clade (electronic supplementary material, table S2, data S1). All but two tumours could be assigned to a clade using this method, confirming that these indeed belong within the previously described DFT1 phylogeny [6,7] (this also confirms that these tumours would be expected to carry the 1311 truncal substitutions genotyped in the false negative screen; figure 1*b*). The two unassigned tumours contained minimal, if any, DFT1 DNA, and were probably composed almost entirely of host tissue. DFT1 clade membership was not, however, reflected in the phylogenetic tree presented by Patton *et al*. [14] (figure 2). Low-coverage tumour sequencing data, such as those of Patton *et al*. [14], are amenable to copy number analysis, although this was not attempted in their study. We identified 155 copy number variants (CNVs) in the tumours from Patton *et al*. [14], 32 of which were phylogenetically informative, and used these as characters to construct a phylogenetic tree (electronic supplementary material, table S4, data S2). Despite its lack of resolution, this tree is consistent with the genotype-assigned DFT1 clades [6,7], and incongruent with the tree presented by Patton *et al*. [14] (figure 2). Thus, the phylogenetic tree presented by Patton *et al*. [14] does not represent the evolutionary history of DFT1.

The errors in the mutation data and phylogenetic tree used by Patton *et al*. [14] in their phylodynamic analysis would be expected to preclude accurate inference of epidemiological parameters. In consequence, estimates of effective reproduction number and effective population size for the DFT1 epizootic reported by Patton *et al*. [14] should, in our opinion, also be considered invalid.

On a broader note, we believe that it is important to clarify that the overall premise of the study by Patton *et al*. [14] was flawed such that, even if suitable genome sequencing depth and accurate mutation calls had been available, meaningful conclusions regarding DFT1 epidemiology were unlikely to be reached. When whole genome sequences are available for mammalian tumours, it is mistaken that phylodynamic methods require screening for ‘measurably evolving portions’ or ‘clock-like genes’. Indeed, the search for ‘clock-like genes’ performed by Patton *et al*. [14] presumably selected for spurious desired signal among thousands of tested genomic interval combinations. After making adjustments for tumours showing evidence of unusual mutational processes, such as the mutational signature of defective DNA mismatch repair observed in DFT1 clade E [7], the entirety of mutations in the nuclear genome is the best possible input for DFT1 mutation rate analysis. Secondly, although repeated independent observation of the same mutation may in rare cases provide evidence for positive selection in DFT1 [7], discovery of such mutations requires ancestral state reconstruction and consideration of copy number states. Searching for the ‘differentiation of SNPs in pairwise comparisons of transmission clusters’ (fig. 4 in Patton *et al*. [14]) is not an appropriate approach for identifying somatic mutations relevant to tumour phenotypes. Finally, and most importantly, the sampling schemes adopted by both Patton *et al*. [14] and our own group [7] introduce biases that preclude accurate inference of epidemiological parameters. In both studies, sparse sampling was performed with the intention of maximising genetic and spatio-temporal diversity. This approach produces phylogenetic trees which are not representative of the epidemiological process (e.g. fig. 1D in Stammnitz *et al*. [7]; note, for instance, the long terminal branches, which reflect purposeful sampling of diversity). The application of phylodynamic models to data that deviate from random sampling requires careful consideration and cautious interpretation [18–21].

Phylodynamic analytical methods have been used effectively to investigate epidemiological processes and demographic histories of a range of pathogens [12,13]. Although Patton *et al*. should be commended for the conceptual expansion of phylodynamics to the study of transmissible cancers, such applications require appropriate data from an unbiased sampling scheme. If suitable samples become available, such methods may, in future, be useful for understanding DFT1 transmission processes. At present, however, there is no evidence that DFT1 has shifted from emergence to endemism, and the outlook for Tasmanian devils remains uncertain.

## 2. Material and methods

### 2.1. Data retrieval and re-alignment

Sequencing data for 51 tumours were obtained from the NCBI Sequencing Read Archive (SRA) [14,22]. Paired-end and single-end reads were aligned to the Tasmanian devil reference assembly mSarHar1.11, GCA_902635505.1 [7], using bwa mem v. 0.7.17 [23], with the parameter settings as specified in Stammnitz *et al*. [7]. Mappings were subsequently sorted and, whenever necessary, merged using samtools v. 1.11 [24].

### 2.2. Tumour sequencing coverage estimation

Mean tumour sequencing coverage for each alignment in Patton *et al*. [14] was calculated using samtools coverage v. 1.11 across chromosomes 1–6 [24].

### 2.3. Purity calculation

To estimate tumour purity, we compared genome-wide sequence coverage depth with that at a 24.7 megabase (Mb) deletion common to all DFT1 tumours (coordinates, chromosome 3:192,161,000–216,866,000 bp relative to mSarHar1.11), as previously described [6,25]. Briefly, sequence depth was counted in 1 kilobase (kb) non-overlapping bins using bedtools coverage v. 2.23.0 [26] across chromosomes 1–6. For each genome, we calculated median coverage across all bins (*C_All_*), as well as median coverage in bins within the chromosome 3 deletion (*C_Del_*). Tumour purity (*ρ*) was then calculated as follows:


$$\rho =1-2\left(\left(\frac{C_{Del}}{C_{All}}\right)-0.5\right)$$

### 2.4. False negative rate estimate

In a separate study, we identified 1311 ‘trunk’ nuclear substitution variants (electronic supplementary material, table S2, data S1) shared by all 78 analysed DFT1 tumours, but absent from all 80 analysed normal Tasmanian devil genomes [7]. We used alleleCount v. 4.0.0 (https://github.com/cancerit/alleleCount; default settings) to determine allele frequencies at each variant position, and defined a variant as ‘present’ if a minimum of three reads supporting the DFT1 allele was found. This represents a slightly less sensitive method of variant genotyping compared with that used in Stammnitz *et al*. [7], with the result that minimal false positive and false negative genotypes are detectable among the tumour and normal samples of Stammnitz *et al*. [7], presented in electronic supplementary material, table S2.

### 2.5. DFT1 clade-specific mutation genotyping

We used alleleCount v. 4.0.0 (https://github.com/cancerit/alleleCount; default settings) to genotype the tumours analysed by Patton *et al*. [14] for subsets of variants unique and universal to each of the six DFT1 clades (electronic supplementary material, table S2; data S1) [6,7], with presence defined as at least 3 supporting reads. A clade assignment was made if at least 3 clade-defining variants supporting a single clade were detected. In one case (T-584236), variants from two clades were identified: 134 of 139 (96%) clade A2 clade-defining variants, along with 35 of 349 (10%) clade C clade-defining variants. We considered it likely that this finding was due to contamination, and assigned this tumour to clade A2.

### 2.6. Substitution density

Patton *et al*. [14] do not provide the coordinates of the mutations identified in their study, precluding direct mutation validation. To gain an understanding of the quality of the Patton *et al*. mutation set [14] we therefore compared the mutation density reported by Patton *et al*. [14] among 51 DFT1 tumours within a restricted genomic interval, with mutation density within a comparable interval among 78 DFT1 tumours reported in Stammnitz *et al*. [7].

Patton *et al*. [14] report mutation density within a selected set of 28 ‘clock-like genes’, spanning 431,608 base pairs (bp), with coordinates reported relative to an earlier Tasmanian devil reference genome, DEVIL7.0 [27]. We retained 15 of these genes, selecting those with 1:1 mapping relationships between DEVIL7.0 and the more recent, chromosome-level reference assembly mSarHar1.11 [7] using the Ensembl Genome Browser (www.ensembl.org), and those with identical HGNC symbols in DEVIL7.0 v. 101 and mSarHar1.11 v. 105 (electronic supplementary material, table S3). The corresponding 670,660 bp interval represents the complete genomic footprint of these 15 genes in mSarHar1.11. We calculated somatic substitution mutation density within this interval among the 78 DFT1 tumours analysed in our separate study, by intersecting these genomic coordinates with the DFT1 somatic substitution mutation list presented in Stammnitz *et al*. [7].

Patton *et al*. [14] state that their final matrix contained ‘2520 parsimony-informative sites, 1893 singletons, 802 doubletons and 2711 variants found in three or more individuals’. We interpreted this to mean that they identified a total of 5406 (1893 + 802 + 2711) variants within the 431,608 bp region.

### 2.7. Mutation rate comparisons

We downloaded viral mutation rate data from [28] (https://github.com/lauringlab/JVI_Gem_2018). Bacterial mutation rates were obtained from [29], retaining estimates from infections among (as opposed to within) hosts, from 13 species. Mammalian somatic mutation rates were obtained from the supplementary materials of [30].

### 2.8. Copy number analysis

Copy number analysis was performed as described in Stammnitz *et al*. [7] using 10 kb bins and restricting inference to autosomes. Normal genomes described in Stammnitz *et al*. [7] were used for normalisation and denoising. Samples that were also analysed in [6] or [7], and found in those studies to be tetraploid, were also considered as tetraploid in this analysis (SAMN14418893 [T-2772], SAMN09242213 [T-487483], SAMN09242224 [T-582387]). Three tumours (SAMN14418891 [T-1560], SAMN14418906 [T-223262], SAMN14418921 [T-695064]) had insufficient sequencing depth, after accounting for purity, for copy number analysis, and were excluded. The 155 detected CNVs (electronic supplementary material, table S4, data S2) were visually validated and genotyped. Seven CNVs known to occur recurrently within DFT1 [6,7] were excluded from tree-building. These included CNVs associated with the unstable ‘marker 5’ chromosome, as well as a CNV locus on chromosome 5 (coordinates, chromosome 5:240,250,001–252,940,000 bp relative to mSarHar1.11) which shows instability in DFT1 [6,7]. A CNV phylogenetic tree was inferred using IQtree [31], allowing the software to select the model best supported by the data (GTR2+FO). Due to sparsity of signal, the tree had several branches of essentially zero length. These were collapsed to multifurcating nodes using the di2multi function of the *Ape* package in R [32]. The CNV tree was compared with the maximum likelihood tree displayed in supplementary figure S3 of Patton *et al*. [14], obtained using the TreeSnatcher software [33], using the tanglegram function of the *dendextend* R package [34] with option ‘fast’ set to TRUE.

## Data Availability

Electronic supplementary material includes supplementary tables S1, S2, S3, S4, S5 and S6, available online at https://doi.org/10.6084/m9.figshare.c.7123847. In addition, supplementary data S1 (DFT1 genotyping tables) and supplementary data S2 (DFT1 copy number profile visualizations) are available at [35].
